# Daylength predominates the bud growth initiation of winter deciduous forest trees in the monsoon region of China

**DOI:** 10.3389/fpls.2023.1327509

**Published:** 2024-01-11

**Authors:** Weiguang Lang, Siwei Qian, Xiaoqiu Chen

**Affiliations:** College of Urban and Environmental Sciences, Laboratory for Earth Surface Processes of the Ministry of Education, Peking University, Beijing, China

**Keywords:** phenology model, bud growth, photoperiod induction, temperature induction, forest trees

## Abstract

Climate warming has induced significant shifts in spring phenology over both temperate and boreal forests. The timing of bud growth resuming from dormancy is crucial for predicting spring phenology. However, the mechanisms by which environmental cues, other than chilling accumulation, initiate bud growth remains unclear. By constructing a revised process-based spring phenology model incorporating photoperiod and temperature triggers of bud growth, we simulated the first leaf unfolding and first flowering dates of four deciduous forest trees during 1981-2014 at 102 stations across China’s monsoon regions. Then, we revealed spatial patterns of the two triggers. Moreover, we compared fitting precision and robustness of the revised model with three mainstream models. Results show that the revised models can effectively simulate all spring phenology time series. Growth initiation of foliar and floral buds was induced by photoperiod lengthening in 80.8% and 77.7% of time series, and by temperature increasing in remaining 19.2% and 22.3% of time series, respectively. The proportions of time series with photoperiod- and temperature-initiated bud growth significantly increase and decrease from northern to southern climatic zones, respectively. Chilling exposure controls the predominant bud growth triggers in different climate zones. Specifically, in regions with long and severe winters where chilling requirement is easily fulfilled, rising temperature in spring alleviates the cold constraint and initiate bud growth. Conversely, in regions with short and mild winters, prolonged daylength in spring compensates the lack of chilling exposure to initiate bud growth. These findings suggest that photoperiod may limit spring phenology response to temperature in low-latitudes. Overall, our model slightly outperforms other models in terms of efficiency, accuracy, and robustness in modeling leaf unfolding and flowering dates. Therefore, this study deepens our understanding of the mechanisms of spring phenology, and improves the predicting capability of spring phenology models in the face of ongoing global warming.

## 1 Introduction

The seasonally alternating growth and dormancy of plants are co-regulated by interaction of endogenous and exogenous drivers, which ensure the organism maximize the resource usage for growth and reproduction and minimize the risk from frost damage (Larcher, 1975; Kramer et al., 1996). The timing of bud growth initiation after dormancy break is a crucial component in modeling the leaf unfolding and flowering. The leafing phenology regulates carbon dioxide, water and energy exchanges between vegetation and atmosphere (Goulden et al., 1996; Myneni et al., 1997; Black et al., 2000; White and Nemani, 2003; Kljun et al., 2007; Barr et al., 2009), while flowering phenology indicates reproductive dynamics of a plant community, and influences changes in interaction relationships among various trophic levels within a food web (Morellato et al., 2016). Nevertheless, the process of determining growth initiation is missing from existing process-based spring phenology models (Chuine et al., 2016), though some efforts were made on fruit trees (e.g., Hillmann et al., 2021). Filling this knowedge gap will be beneficial for better understanding mechanisms of spring phenological occurrence timings, and improving phenological prediction accuracy under future warming scenarios.

Many studies have revealed that buds of temperate deciduous trees usually experience endodormancy and ecodormancy stages during the wintering period (Lang et al., 1987; Anderson et al., 2010; Cooke et al., 2012). Specifically, buds enter the endodormancy stage induced by endogenous factors along with the decrease of photoperiod and temperature in previous autumn. During the endodormancy stage, buds may respond to low but non-freezing temperature (chilling temperatures) within a specific range (e.g., 0 - 5°C or 0 - 12°C)(Myking and Heide, 1995; Ghelardini et al., 2010; Baumgarten et al., 2021; Danieli et al., 2023). Sufficient exposure to chilling temperatures break the endodormancy (Horvath et al., 2003; Cooke et al., 2012). Afterward, buds enter the ecodormancy stage immediately or after a period of queisence. Some forest tree species need an additional weather independent photoperiod signal to effectively advance the transition from the endodormancy to the ecodormancy (Basler, 2016). At the onset of the ecodormancy stage, buds initiate growth and become responsive to the increasing forcing temperature (Penfield, 2008), but significant changes in bud appearance morphology are often not visible. The daily bud growth rate is promoted by forcing temperatures (Cooke et al., 2012). When a certain amount of forcing temperature accumulation (forcing requirement) is satisfied, the ecodormancy state releases, and then spring phenological events occur (Chuine, 2000). There are yet no well-defined physiological or molecular markers to clearly separate the two dormancy stages for forest trees (Basler, 2016). In fruit trees as well, there are few studies dedicated to identifying different dormancy stages. One example is to use the state of pistils as a marker (Wang et al., 2020).

The timings of bud growth initiation were normally identified through destructive measurements of bud primordium lengths or derivations from the result of manipulative experiments (Cannell and Smith, 1983; Cooke et al., 2012; Sutinen et al., 2012; Hänninen et al., 2018). These experiments hypothesized that the forcing requirements to break ecodormancy decrease with increase of the chilling days before bud growth initiation. Once the chilling accumulation exceeds a threshold (chilling requirements), the forcing requirements keep constant (Hänninen et al., 2018). Researchers determined the minimum chilling requirements by identifying the inflection point, at which the forcing requirements transition from increase to remaining constant. The date of bud growth initiation is then deduced from the inflection point (Hänninen et al., 2018). Some studies showed that temperate and boreal trees (e.g., *Picea glauca*) can initaite bud growth and enter into the ecodormancy stage, once the endodormancy terminates (Cooke et al., 2012). However, for some temperate deciduous species (e.g., leafy spurge plants), buds cannot enter into the ecodormancy period immediately after endodormancy releases under current climate conditions. Instead, buds would keep the quiescence state until the local temperatures are warm enough (Horvath et al., 2010; Chao et al., 2015) or the daylengths reach a threshold (Wu, 2003; Linkosalo and Lechowicz, 2006) to initiate their growth. Besides, even the chilling requirements could never be satisfied, the bud growth may also be initiated under specific conditions. For example, high forcing temperature may compensate the insufficient chilling accumulation and initiate the bud growth (Shirazi, 2003). The factors initiating bud growth may diverse among local environmental conditions and species (Zohner et al., 2016). Nevertheless, either manipulative experiments conducted mainly on fruit trees (Dennis, 2003; Chmielewski et al., 2017), or destructive measurements of bud primordium lengths (Cannell and Smith, 1983; Sutinen et al., 2012) are restricted to specific sites and species. It is still challenging to identify the timings of bud growth initiation at large scales.

Process-based spring phenology models track the progress of bud dormancy and growth through portraying physiological responses of buds to environmenal changes. Based on the highly positive correlation between thermal accumulation and length of the bud primordium (Cannell and Smith, 1983; Sutinen et al., 2012), researchers have constructed several one-phase models to simulate the entire progress from bud growth initiation to spring phenology occurrence. In these models, the bud development states are represented by accumulation of bud growth rate estimated by a linear or nonlinear function of daily mean air temperature (Chuine, 2000). Up to now, one-phase models have been effectively applied to simulate leaf unfolding and flowering dates worldwide (Cannell and Smith, 1983; Murray et al., 1989; Hänninen, 1990; Kramer, 1994; Chuine, 2000; Chuine et al., 2000; Linkosalo et al., 2008; Richardson and O’Keefe, 2009; Xu and Chen, 2013; Luo et al., 2014; Chen et al., 2017; Zhang et al., 2022; Zhang et al., 2023). However, the timing of bud growth initiation was determined either by thresholds of forcing temperature (Cannell and Smith, 1983) or a prescribed date, i.e., 1^st^ January (Chuine, 2000). By contrast, the timing of bud growth initiation is usually set to be the endodormancy break date in the two-phase models (Chuine, 2000). This prescribed setting neglected the process of environmental factors initiating bud growth, and reduced the physiological significance and simulation accuracies of process-based models (Chuine et al., 2016; Hänninen et al., 2018). Whether and how the environmental factors initiate bud growth remain unclear.

In this study, we determined the potential optimum timing of bud growth initiation according to priority response of bud growth process to temperature or photoperiod, and integrated the timing into the one-phase models to improve the simulation and prediction effectiveness of spring phenology. Moreover, we analyzed the spatial pattern of temperature and photoperiod inducing bud growth initiation for overwinter deciduous trees across the eastern monsoon region of China. We aimed to address the following scientific questions: (1) Can temperature and photoperiod thresholds indicate the bud growth initiation of deciduous forest trees and enhance the model effectiveness in simulating spring phenology? (2) What are the spatial patterns of two triggers (temperature and photoperiod) at multiple and single species levels? and (3) What is the climatic attribution of the spatial differentiation of two triggers?

## 2 Materials and methods

### 2.1 Study area and tree species

**Figure 1:**
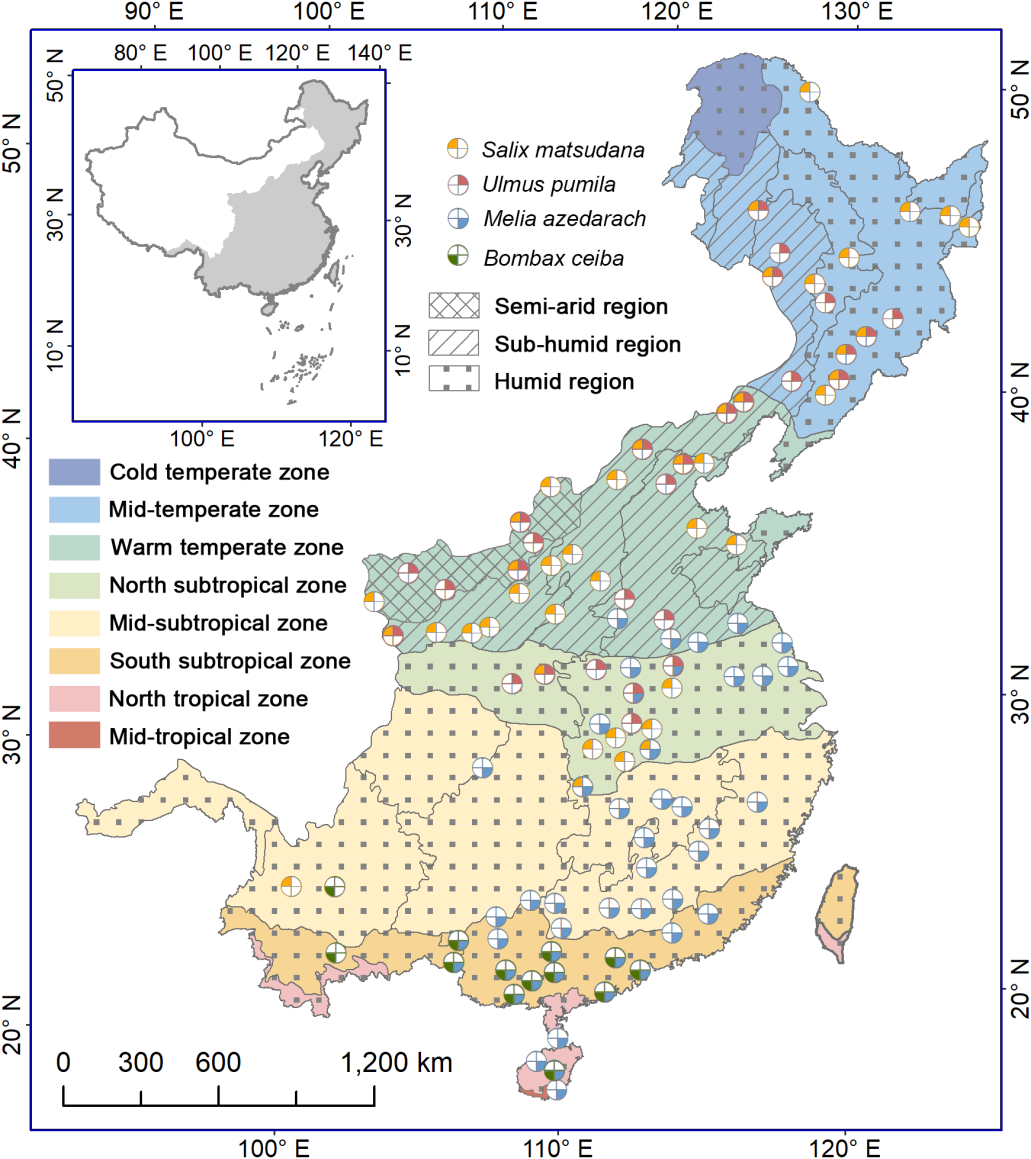
Distribution of phenological stations for four indicative tree species in the monsoon region of China. The colors of the zones denote different climatic zones, while the filled patterns represent regions with varying humidity levels. The colors and positions of the four sectors represent the following species: *Salix matsudana*, *Ulmus pumila*, *Melia azedarach* and *Bombax ceiba*. Starting from the top left corner and moving clockwise, each sector corresponds to one of the mentioned species. A white sector denotes the lack of observed data for the corresponding tree species at the station.

The study area covers the eastern monsoon region of China, ranging from 91.5°E to 135.1°E and from 18.2°N to 53.6°N. The eastern part of the region consists of plains, hills and low mountains, and the western part is dominated by mountains. The elevation rises from 0 m in the eastern coast to 5200 m in the western interior. Under influence of the Pacific summer monsoon, the annual precipitation decreases roughly from 2000 mm in the southern part to 400 mm in the northern and northwestern parts. The precipitation concentrates mostly in summer (from June to August), accounting for more than 40% of the annual total precipitation. The annual mean air temperature reduces from 25°C in the southmost to -9°C in the northmost. According to similarity of hydrothermal conditions, 7 climatic zones and 10 eco-geographical regions have been divided, namely, cold temperate humid region, middle temperate humid and sub-humid regions, warm temperate humid, subhumid and semiarid regions, north subtropical humid region, middle subtropical humid region, south subtropical humid region, and north tropical humid region (Zheng, 2015) (**Figure 1**). The vegetation types include forests in mountainous areas (such as cold temperate coniferous forest, temperate broadleaved and coniferous mixed forest, warm temperate deciduous broadleaved forest, subtropical evergreen broadleaved forest, north tropical seasonal rainforest and rainforest), and cultivated vegetation on the plains and hills (Wu, 1980).

We selected four common deciduous trees as the indication species, namely, *Salix matsudana*, *Ulmus pumila*, *Melia azedarach* and *Bombax ceiba*. *S. matsudana* and *U. pumila* are native temperate species with high frost and drought tolerance, and distributed mainly from the middle temperate zone to the north subtropical zone. *M. azedarach* and *B. ceiba* are tropical origin species with low frost resistance (Chen et al., 2017). Currently, the former is distributed mainly from the southern part of the warm temperate zone to the north tropical zone, and the latter appears only in the south subtropical zone and north tropical zone. All the four tree species show an annually recurring growth and dormancy cycle. Thus, these trees are suitable for examining timings and triggers of bud growth initiation, and their influence on simulation effectiveness of spring phenology.

### 2.2 Phenological and meteorological data

Plant phenological data were acquired from the China Meteorological Administration (Chen, 2013). The phenological observations are implemented normally every other day by professionals following uniform observation criteria (China Meterological Administration, 1993). More than three individual trees were selected as fixed observation objects for each species at a given location. Here we selected first leaf unfolding and first flowering dates of the four tree species to conduct this study. According to phenological observation criteria, the first leaf unfolding date is defined as the day when about 5% of leaves on a tree have emerged and started to unfold, observed on more than half of the individuals, while the first flowering date is identified as the day when about 5% of flower buds are fully opened on more than half of the observed trees (China Meterological Administration, 1993). We checked the first leaf unfolding and first flowering data of each species at each site and removed outliers that were beyond two standard deviations from the mean value. Additionally, we excluded time series less than 10 years. Finally, we obtained a dataset containing 130 time series for first leaf unfolding dates and 130 time series for first flowering dates at 102 phenological stations, including 42 times series for *S. matsudana*, 28 times series for *U. pumila*, 47 times series for *M. azedarach* and 13 times series for *B. ceiba* (**Figure 1**).

Daily mean air temperature data at 102 national meteorological and climatological stations from 1980 to 2014 were acquired from the China Meteorological Data Service Center (http://data.cma.cn/). All the national meteorological and climatological stations are located at or nearby the corresponding phenological stations within the maximum distance of 5 km. The daily mean air temperature data, which have been examined and verified by the China Meteorological Information Center, were used to calibrate and validate the process-based models. In the further analyses, spring temperature variation in a location is calculated as the multiyear mean value of the standard deviation of the detrended daily mean air temperature during 60-day period preceding the mean phenological date. Winter temperature denotes the multiyear mean temperature from previous December to February, while the winter duration was defined as the number of days with mean temperature lower than 5°C from 1st November of the previous year to the multiyear mean first leaf unfolding or first flowering dates (Hunter and Lechowicz, 1992; Zohner et al., 2016). Daylengths at each phenological station were calculated based on the latitude at a given station and day of year (DOY) according to the modified Schoolfield’s equations (Forsythe et al., 1995). The daylengths increase from 11.0 h to 13.2 h at the southernmost site (site ID: 59954, **Supplementary Table 1**) and from 7.8 h to 16.7 h at the northernmost site (site ID: 50353, **Supplementary Table 1**) from winter solstice to summer solstice.

### 2.3 Revised process-based spring phenology model

We revised the widely used UniForc model (Chuine, 2000) through determining temperature and photoperiod thresholds of bud growth initiation, and termed it as the TPForc model. The basic hypothesis of the TPForc model is that buds initiate growth when favorable environmental conditions achieve, namely, either temperature or daylength exceed the respective threshold. Afterward, the daily bud growth rate (*R_f_*) is influenced by forcing temperature, and changes in a sigmoid function as daily mean air temperature (*T_t_*) rises (Equation 1) (Chuine, 2000). The bud growth state (*S_f_*) is the accumulation of daily bud growth rate (*R_f_*) from the bud growth start date (*D_start_*) to any dates before spring phenology occurs. When the bud growth state (*S_f_*) reaches the critical value (F*) on date *D_s_*, the spring phenology (leaf unfolding/flowering) occurs (Equation 2).


(1) $$R_{f}(T_{t})=\frac{1}{1+e^{f_{a}(T_{t}-f_{b})}}$$

(2) $$S_{f}=\sum_{t=D_{start}}^{D_{s}}R_{f}(T_{t})=F^{*}$$

where *T_t_* is daily mean air temperature on the date *t*. *f_a_* and *f_b_* are parameters controlling the response of bud growth rate to temperature (*f_a_* < 0 and *f_b_* > 0). The bud growth start date (*D_start_*) is determined in two ways. If bud growth initiation is induced by temperature rising, *D_start_* is the first day when daily mean air temperature is higher than a temperature threshold (*T_start_*) after the coldest date in the preceding winter (i.e., January 20^th^ in the current year) (Equation 3). If bud growth initiation is triggered by photoperiod lengthening, *D_start_* is the first day when daylength is longer than a photoperiod threshold (*P_start_*) after the shortest daylength date (i.e., winter solstice date in the previous year) (Equation 4).


(3) $$D_{start}=\text{firstday when }T_{t}>T_{start}$$

(4) $$D_{start}=\text{firstday when }P_{t}>P_{start}$$

where *T_start_* is the temperature threshold. *P_start_* is the daylength threshold. The model therefore contains two sub-models portraying the pathways with temperature-initiated bud growth (TPForc_t_ model) and photoperiod-initiated bud growth (TPForc_p_ model), respectively. Both sub-models contain four fitted parameters: *T_start_*/*P_start_*, *f_a_*, *f_b_* and F^*^.

### 2.4 Model calibration and validation

The optimal parameter combinations of the TPForc_t_ and TPForc_p_ models in fitting each site-species-phenophase time series were detemined by the minimum root mean squared error (RMSE) (Equation 5) between observed and fitted time series through the simulated annealing algorithm of Metropolis (Chuine et al., 1998). Then, the optimum local species-specific phenology model was selected according to the lower value of RMSEs between the TPForc_t_ and TPForc_p_ models. Moreover, the simulation effectiveness of each optimum model was assessed using Nash-Sutcliffe Efficiency (NSE) (Equation 6) by comparing with the null model (namely, mean occurrence dates of spring phenology) (Nash and Sutcliffe, 1970). A positive NSE value indicates that the model explains more spring phenology variance than the null model. The larger the positive NSE value (between 0 and 1), the higher the model effectiveness. In contrast, a negative NSE value represents that the model performs worse than the null model.


(5) $$RMSE=\sqrt{\frac{\sum_{i=1}^{n}{\left(O_{i}-F_{i}\right)}^{2}}{n}}$$

(6) $$NSE=1-\frac{\sum_{i=1}^{n}{\left(O_{i}-F_{i}\right)}^{2}}{\sum_{i=1}^{n}{\left(O_{i}-\bar{O}\right)}^{2}}$$

where *O_i_* and *F_i_* are the observed and fitted spring phenology date in year *i*, respectively. *Ō_i_* is the mean observed spring phenology date. *n* is the number of years.

We employed the leave-one-out cross-validation analysis to evaluate the ability of the TPForc model in predicting first leaf unfolding and first flowering dates in years beyond the period of model fitting (Lang et al., 2019). Specifically, for a *n*-year phenological time series, each *n*-1 years’ phenological dataset were sequentially fitted (calibration) by the TPForc model and the fitted parameters were used for predicting the phenology date in the remaining year (validation). This process was repeated *n* times, so that phenological date of each year was included in the validation dataset exactly once. The validation error was measured by validation RMSE (VRMSE) between observed and predicted phenological dates across the *n* years. This cross-validation is appropriate for datasets with small sample sizes.

### 2.5 Comparison among the TPForc model and other models

To validate reliability of the TPForc model, we compared its performance in modeling spring phenology with those of some other existing one-phase spring phenology models (i.e., UniForc model, Chuine, 2000; Photothermal model, Masle et al., 1989; Basler, 2016; M1 model, Blümel and Chmielewski, 2012) (**Supplementary Text**). NSE, RMSE and correlation coefficient between observed and simulated phenological time series were used to measure effectiveness and accuracy of these models. In addition, the small-sample corrected Akaike Information Criterion (AICc) was employed to evaluate the parsimony and efficiency of these models (Equation 7):


(7) $$AICc=n\times \ln(\frac{\sum_{i=1}^{n}{\left(O_{i}-F_{i}\right)}^{2}}{n})+\frac{2n(k+1)}{n-k-2}$$

where *k* is the number of parameters, the other variables are the same as in Equation 5. AICc can effectively balance simulation accuracy against overparameterization. The model with the lowest AICc is usually considered as the best model with high accuracy and less parameters for a given dataset.

## 3 Results

### 3.1 Local species-specific optimum models and their performances

We fitted the TPForc model using 260 time series for first leaf unfolding and first flowering dates of the four tree species during 1981-2014, and selected the optimum models for each time series (**Supplementary Table 1**). The results show that all optimum models are more effective than null models (NSE>0). NSEs are larger than 0.3 for 90% of leaf unfolding time series and 82.3% of flowering time series. For the 130 first leaf unfolding time series, the TPForc_t_ and TPForc_p_ models account for 19.2% and 80.8%, respectively, while for the 130 first flowering time series, the two types of optimum models occupy 22.3% and 77.7%, respectively. In terms of interspecific differences, percentages of the optimum TPForc_t_ model in fitting first leaf unfolding and first flowering dates are larger for native temperate species (*S. matsudana* and *U. pumila*) than for tropical origin species (*M. azedarach* and *B. ceiba*), whereas percentages of the optimum TPForc_p_ model in fitting first leaf unfolding and first flowering dates are larger for tropical origin species than for native temperate species (**Table 1**). Therefore, bud growth initiation is predominantly induced by photoperiod lengthening, especially for the tropical origin species.

**Table 1:** Comparison of simulation and prediction accuracy of optimum models.

	First leaf unfolding				First flowering			
	*Salix matsudana*	*Ulmus pumila*	*Melia azedarach*	*Bombax ceiba*	*Salix matsudana*	*Ulmus pumila*	*Melia azedarach*	*Bombax ceiba*
Percentage of TPForc_t_	31	21.4	10.6	7.7	42.9	28.6	6.4	0
Percentage of TPForc_p_	69	78.6	89.4	92.3	57.1	71.4	93.6	100
Mean *RMSE* (d)	4.5	4.3	5.3	6.8	5.4	6.6	4.6	9.1
Percentage of optimum models (*RMSE<* 6d)	88.1	82.1	57.4	38.5	69	42.9	76.6	0
Percentage of optimum models (*p*< 0.05)	92.9	89.3	95.7	100	95.2	89.3	91.5	92.3
Mean V*RMSE* (d)	5.8	5.2	6.9	8	7.2	8.3	6	12.6
Percentage of optimum models (V*RMSE<* 6d)	52.4	75	46.8	30.8	38.1	21.4	55.3	7.7

The mean simulation error (RMSE) for all time series is 5.4 days, with 5.0 days for first leaf unfolding and 5.7 days for first flowering. Optimum models with RMSE smaller than 6 days account for 72.3% for first leaf unfolding and 59.2% for first flowering. The simulated first leaf unfolding and first flowering dates correlate significantly (p<0.05) and positively with observed first leaf unfolding and first flowering dates in 96.9% and 95.4% of time series. Regarding interspecific differences, the simulated RMSEs of first leaf unfolding dates for native temperate species (4.5 days for *S. matsudana* and 4.3 days for *U. pumila*) are much smaller than those of tropical origin species (5.3 days for *M. azedarach* and 6.8 days for *B. ceiba*). Percentages of optimum models with simulated RMSE< 6 days for native temperate species (88.1% for *S. matsudana* and 82.1% for *U. pumila*) are markedly larger than those for tropical origin species (57.4% for *M. azedarach* and 38.5% for *B. ceiba*). In contrast, percentages of significant and positive correlation coefficients between observed and simulated first leaf unfolding dates for native temperate species (92.9% for *S. matsudana* and 89.3% for *U. pumila*) are smaller than those for tropical origin species (95.7% for *M. azedarach* and 100% for *B. ceiba*). However, neither RMSEs nor correlation coefficients between observed and simulated first flowering dates show above interspecific differences (**Table 1**).

The leave-one-out cross-validation analysis shows that the mean validation error (VRMSE) for the total 260 time series is 7.0 days, which is 1.6 days larger than the average simulation error (RMSE). The mean VRMSEs are 6.4 days and 7.6 days for first leaf unfolding and first flowering, respectively. At species levels, the VRMSE ranges from 5.2 days (for first leaf unfolding of *U. pumila*) to 12.6 days (for first flowering of *B. ceiba*). Considering different types of species, the VRMSEs of predicting native temperate species first leaf unfolding dates are significantly smaller than those of predicting tropical origin species first leaf unfolding dates. Similarly, percentages of optimum models with VRMSE< 6 days for predicting native temperate species first leaf unfolding dates (52.4% for *S. matsudana* and 75% for *U. pumila*) are obvious larger than those for predicting tropical origin species first leaf unfolding dates (46.8% for *M. azedarach* and 30.8% for *B. ceiba*). However, no such differences were detected in predicting first flowering dates (**Table 1**). Generally speaking, the TPForc model has high accuracy and robustness in simulating and predicting first leaf unfolding and first flowering dates of the four tree species across the eastern monsoon region of China.

### 3.2 Spatial pattern of local optimum models

**Figure 2:**
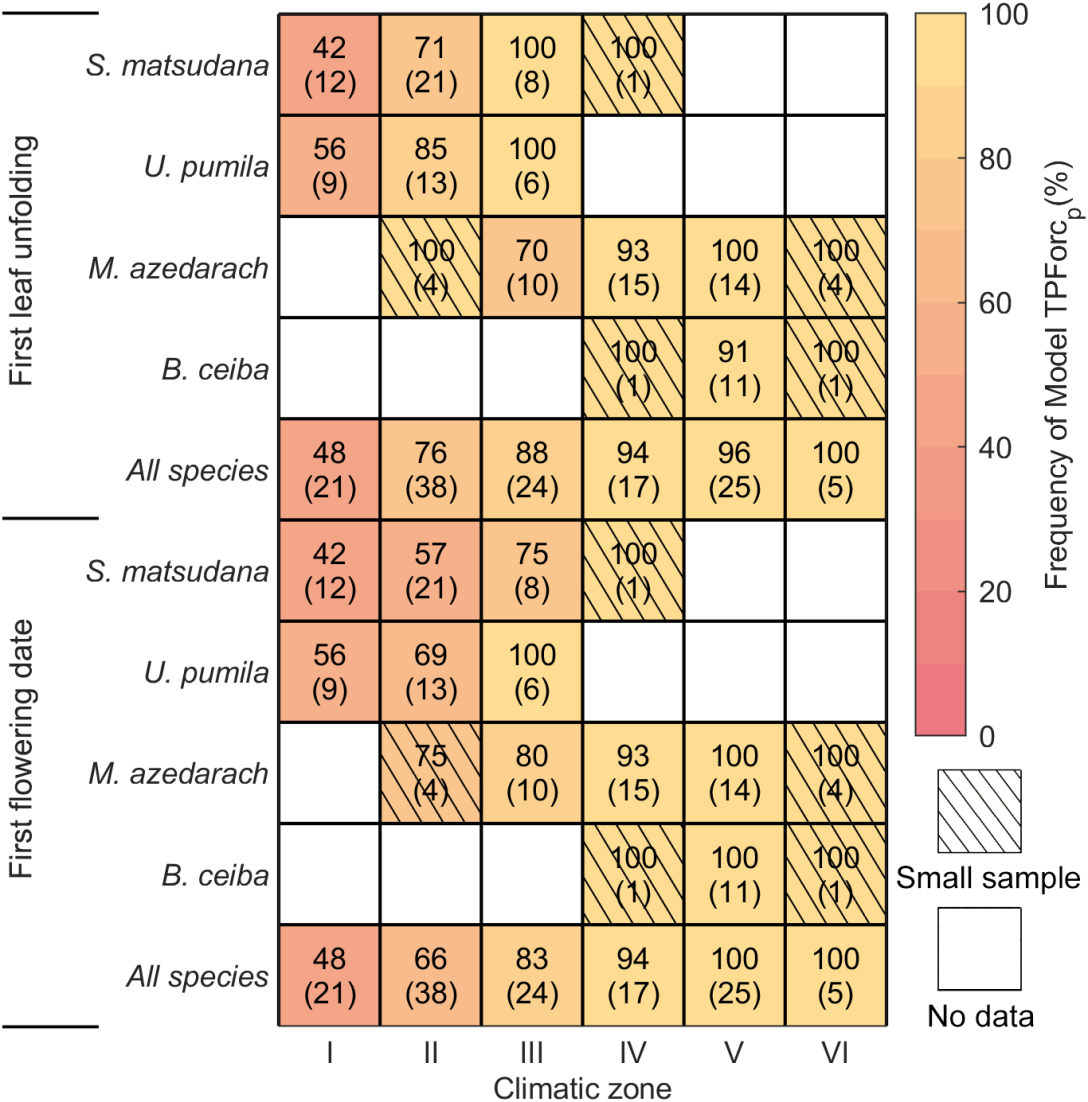
Frequencies of the optimum model TPForc_p_ in different climatic zones for first leaf unfolding and first flowering of four tree species. The numbers in the square show the percentage of the TPForc_p_ model accounting for the optimum models, while the numbers in the bracket showed sample sizes, i.e., the number of the time series with available phenological data. The samples with small sizes (n ≤ 5 time series) have been shaded. Climatic zones I, II, III, IV, V and VI denote middle temperate zone, warm temperate zone, north subtropical zone, middle subtropical zone, south subtropical zone and north tropical zone, respectively.

To detect the spatial differentiation of the two triggers inducing bud growth initiation, we computed the frequency of the TPForc_p_ and TPForc_t_ models accounting for all local species-specific optimum models in each of the six climatic zones (i.e., middle temperate zone, warm temperate zone, north subtropical zone, middle subtropical zone, south subtropical zone and north tropical zone) and sorted them from north to south. The frequencies of the TPForc_p_ and TPForc_t_ models exhibit significant spatial gradients when considering all species together (**Figure 2**). For first leaf unfolding modeling, frequency of the TPForc_p_ model increases from 47.6% in the middle temperate zone to 96.0% in the north tropical zone, while that of the TPForc_t_ model declines from 52.4% to 4% (**Figure 2**). Similar spatial patterns were detected for first flowering modeling, namely, frequency of the TPForc_p_ model increases from 47.6% to 100%, while that of the TPForc_t_ model declines from 52.4% to 0% (**Figure 2**). On the species level, besides *B. ceiba* that distributes mainly in the south subtropical zone and lacks sufficient data to assess its spatial pattern, frequencies of the TPForc_p_ and TPForc_t_ models for both phenophases of the other three tree species show similar spatial patterns along with the geographic-climatic zones from north to south (**Figure 2**).

### 3.3 Comparison between the TPForc model and existing mainstream models

**Figure 3:**
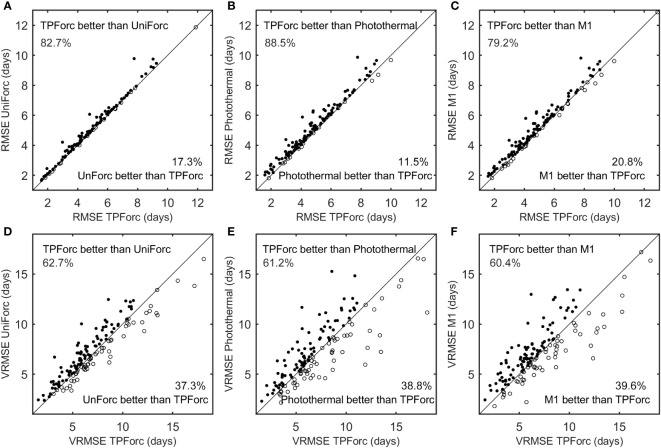
Comparison of simulation and validation performances among the four process-based models. **(A)** simulation root mean square error (RMSE) between the TPForc and UniForc models; **(B)** RMSE between the TPForc and Photothermal models; **(C)** RMSE between the TPForc and M1 models; **(D)** validation root mean square error (VRMSE) between the TPForc and UniForc models; **(E)** VRMSE between the TPForc and Photothermal models **(F)** VRMSE between the TPForc and M1 models.

We compared performances in simulation and validation of the optimum TPForc model and three commonly used one-phase process-based models (namely, the UniForc model, the Photothermal model and the M1 model) using the 260 time series of first leaf unfolding and first flowering dates. The mean simulation NSEs of the TPForc model and the UniForc model are positive for all the 260 time series with the mean value of 0.54 and 0.51, respectively, whereas the NSEs of the Photothermal model and the M1 model are positive for 99.6% of time series with the mean value of 0.47 and 0.49 (**Table 2**). The mean simulation AICc of the TPForc model is smaller than those of the UniForc model (AICc_TPForc_-AICc_Uniforc_=-1.5) and the M1 model (AICc_TPForc_-AICc_M1_=-2.5), but slightly larger than that of the Photothermal model (AICc_TPForc_-AICc_Photothermal_=0.7) (**Table 2**). However, the TPForc model has the same or smaller AICc (namely, higher parsimony and efficiency) than the UniForc model, the Photothermal model and the M1 model in 100%, 55.4% and 95.4% of time series, respectively. The average simulation RMSE (5.4 days) of the TPForc model for the 260 time series is 0.2-0.3 days smaller than those of the other three models (**Table 2**), and the TPForc model exhibits higher simulation accuracy (smaller RMSE) than the UniForc model, the Photothermal model and the M1 model in 82.7%, 88.5% and 79.2% of time series, respectively (**Figures 3A–C**). In addition, the average correlation coefficient between simulated and observed time series for the TPForc model is higher than those of the other three models. The significant (p< 0.05) correlation coefficients between simulated and observed time series for the TPForc model account for 96.2% of the 260 time series, which is also higher than percentages for the other three models (90.4-93.5%) (**Table 2**).

**Table 2:** Comparison of simulation accuracy between TPForc and existing models.

Model	Mean *RMSE* (d)	Mean NSE	Mean correlation coefficient	Percentage of time series (p< 0.05)	Mean AICc	Mean *VRMSE* (d)
TPForc	5.4	0.54	0.73	96.2	90.3	6.9
UniForc	5.6	0.51	0.71	90	91.8	7.4
Photothermal	5.7	0.47	0.68	90.4	89.6	7.3
M1	5.7	0.48	0.69	90.8	92.8	7.4

The models’ robustness comparison shows that validation errors (VRMSE) of the TPForc model are smaller than those of the UniForc model, the Photothermal model and the M1 model in 62.7%, 61.2% and 60.4% of time series, respectively (**Figures 3D–F**). The average VRMSE of the TPForc model is 0.4-0.5 days smaller than those of the other three models (**Table 2**), indicating that the TPForc model has a slightly higher robustness than the other three models in predicting spring phenology. Overall, the TPForc model has higher effectiveness, efficiency, accuracy, and robustness than the other three models, though the improvement in simulation accuracy for certain time series is marginal.

## 4 Discussion

### 4.1 Attributions of bud growth initiation triggers

This study shows that bud growth initiation of four indicative tree species is induced predominantly by prolonged daylength (74.4% of total time series), and less by increased temperature (25.6% of total time series). These two triggers initiating bud growth have been validated by numerous manipulative experiments, that is, buds need a fixed threshold of either photoperiod or temperature to initiate growth (Heide, 1993a, Heide, 1993b; Linkosalo and Lechowicz, 2006; Caffarra and Donnelly, 2011; Flynn and Wolkovich, 2018). Moreover, we found a rough spatial tendency in the triggers that initiate the bud growth among species. Namely, the proportion of phenological time series with bud growth triggered by photoperiod lengthening shows an increasing tendency from north to south, but by temperature increment indicates a decreasing tendency, which is limited by species distribution ranges. This spatial pattern of the two triggers of bud growth initiation detected by model fitting is in agreement with the spatial pattern of bud growth response to photoperiod and temperature based on experimental findings (Borchert and Rivera, 2001; Vitasse and Basler, 2013; Way and Montgomery, 2015; Zohner et al., 2016). Specifically, budburst is regulated by temperature or photoperiod in temperate species (Heide, 1993a; Heide, 1993b; Körner and Basler, 2010; Basler and Körner, 2012; Laube et al., 2014), but by photoperiod in tropical species (Borchert and Rivera, 2001).

**Figure 4:**
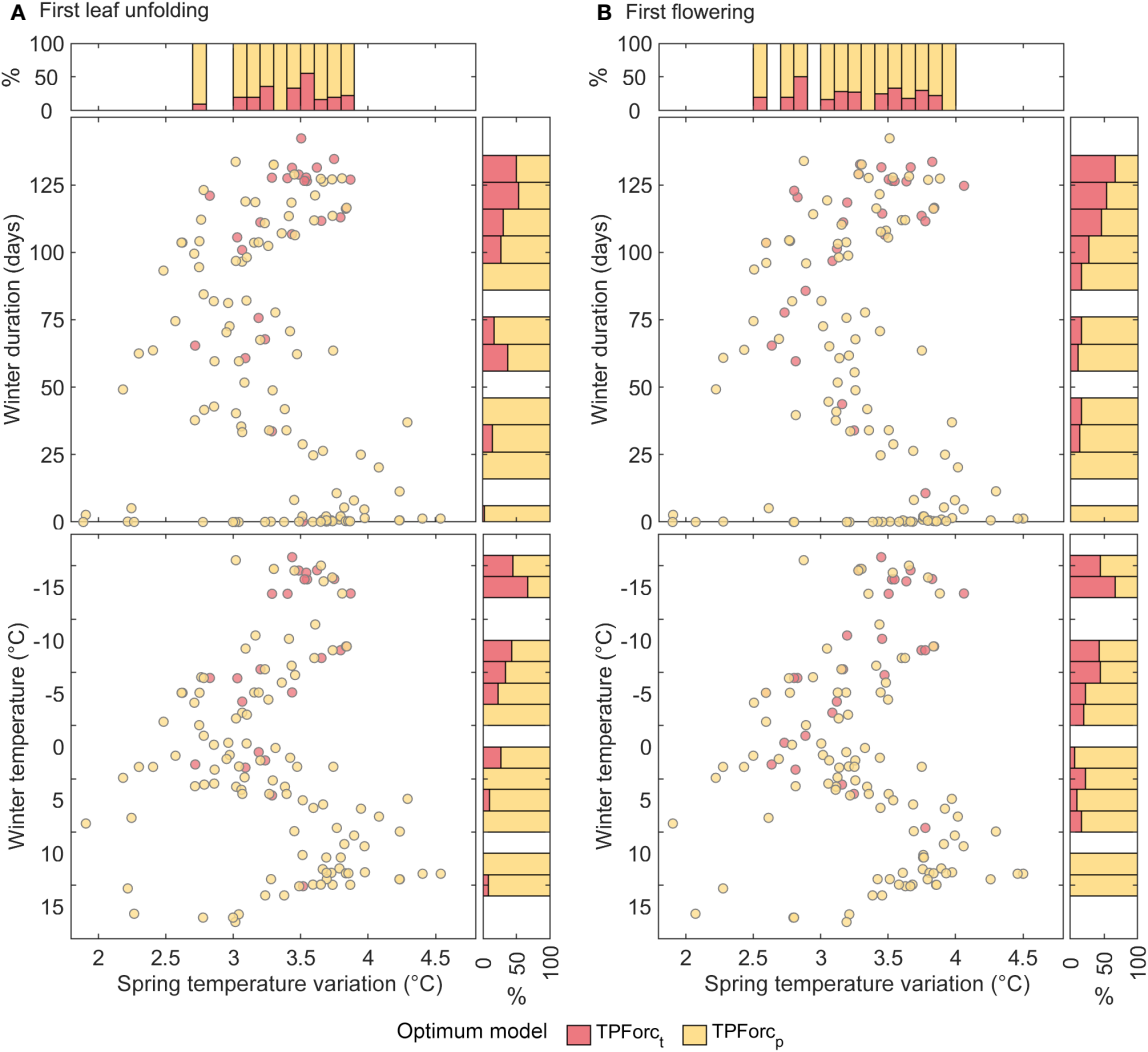
Dependence of optimum model types for first leaf unfolding **(A)** and first flowering **(B)** on spring temperature variation and winter duration and winter temperature. Spring temperature variation is the multiyear mean standard deviation of daily mean air temperature during 60-day period preceding the mean spring phenology date. The winter duration was defined as the number of days with mean temperature lower than 5°C from 1^st^ November of the previous year to the mean first leaf unfolding or first flowering dates. Winter temperature denotes the multiyear mean temperature from previous December to February. The upper panel denotes the proportion of the two models within each 0.1°C bin of temperature standard deviation, while the right panel denotes the proportion of the two models within each 10 days bin of winter duration. The bins containing less than 5 sites were excluded.

Several hypotheses can be used to explain the above spatial pattern of bud growth response to photoperiod. The first hypothesis is that photoperiod, as a stable signal, can help buds to escape frost injuries for developing leaves/flowers by timing bud growth appropriately. If this is the case, photoperiod should be especially important in regions with unpredictable frost events, such as regions with highly interannual variability of spring temperatures (Wang et al., 2014). Thus, we estimated the frequency of the temperature-initiated bud growth model (TPForc_t_) and photoperiod-initiated bud growth model (TPForc_p_) with different degrees of spring temperature variation. However, we did not find a clear dependence of optimum model proportions on spring temperature variations (**Figure 4**).

The second hypothesis is that reducing chilling days may amplify the dependence of overwintering buds on photoperiod (Zohner et al., 2016). To validate this hypothesis, we calculated the multiyear mean winter temperature (December to February) and the mean winter duration for each site. The results show that bud growth initiation of trees relies on either photoperiod or temperature in areas with long and severe winters, whereas bud growth initiation depends mainly on photoperiod in areas with short and mild winters (**Figure 4**). This spatial pattern can be elucidated by the compensatory effect of increasing photoperiod on unfulfilled chilling requirements (Caffarra and Donnelly, 2011; Zohner and Renner, 2015). An experimental study emphasized the significance of adequate chilling exposure for bud growth in subtropical and tropical zones of China (Du et al., 2019), while a process-based modeling highlighted the challenges of unfulfilling chilling requirements for tropical trees due to high winter and spring temperatures (Chen et al., 2017). In such circumstances, the increased daylength in spring as a predominant cue can compensate the insufficient chilling exposure and trigger bud growth initiation. In temperate regions however, plants undergo sufficient chilling accumulation during long and severe winters (Luo et al., 2014). Thus, bud growth initiation is primarily controlled by forcing temperature in spring, while photoperiod has relatively little effect on bud growth initiation. The dependency of bud growth initiation triggers on winter duration and winter temperature implies that the effect of photoperiod on spring phenology may enhance with decline in chilling days under global warming (Körner and Basler, 2010).

### 4.2 Comparison of performance among different spring phenology models

Biological models are generally assessed by three criteria: reality, accuracy, and generality (Levins, 1968). Reality refers to the reasonability of mechanisms behind the model. Our revised spring phenology model (the TPForc model) assumes that the timing of bud growth initiation may indicate start of ecodormancy, and influence performances of spring phenology simulation. The underlying mechanism for constructing the new model was acquired from results of some manipulative experiments, namely, prolonged daylength and increased temperature are key factors for triggering endodormancy release and bud growth initiation (Ikemoto, 1961; Jensen and Gatherum, 1965; Horvath et al., 2003; Singh et al., 2017), and the timimg of bud growth initiation plays a major but poorly defined role in modeling spring phenology (Chuine et al., 2016). In terms of accuracy, the average fitting error of the TPForc model is 0.2-0.3 days smaller than those of the other three models. The average fitting NSE and average correlation coefficient between observed and simulated spring phenological dates for the TPForc model are larger than the other three models (**Table 2**), whereas the TPForc model redundancy (AICc) is the second smallest one in the four models (**Table 2**). Moreover, the average validation error of the TPForc model is 0.4-0.5 days smaller than those of the other three models (**Table 2**). Regarding generality, the TPForc model can effectively simulate and predict first leaf unfolding and first flowering dates of the four tree species from temperate to tropical zones across the eastern monsoon region of China. The model applicability worldwide needs to be validated in future studies. Therefore, the TPForc model comprehensively improves simulation and prediction performances of process-based spring phenology models by incorporating the threshold and its timing of photoperiod or temperature initiating bud growth. Despite the improvement in simulation accuracy for certain time series is marginal, this revised model provides a new insight in better capturing ecophysiological responses of plants to environmental cues. This capability is critical for accurately predicting spring phenology dates under future climate change scenarios.

The Photothermal model and the M1 model assume that daylength may influence the growth and reproduction processes after bud growth initiation (Masle et al., 1989; Blümel and Chmielewski, 2012; Basler, 2016). However, both models performed worse than the forcing temperature-driving UniForc model and the TPForc model. This does not support the above assumption of daylength controls on daily bud growth rate. Meanwhile, the TPForc model shows clear advantages compared with the UniForc model. Our study highlights that incorporating the timing of bud growth initiation induced by photoperiod or temperature into model can lead to more accurate and reliable simulation and prediction of spring phenology.

In this study, we revised the widely-used one-phase UniForc model by determining potential timing of bud growth initiation, and simulated the first leaf unfolding and first flowering dates of four tree species over the eastern monsoon region of China. The extension of daylength is the main trigger of bud growth initiation, surpassing the increase of temperature. As regional temperature increases from middle temperate zone to tropical zone, roles of daylength induction to bud growth initiation become stronger but those of temperature induction become weaker. Further analysis indicates that chilling exposure controls predominant bud growth initiation triggers in different climate zones. The new model displays higher efficiency, accuracy and robustness than existing mainstream models, and provides new insights for understanding mechanisms of leaf unfolding and flowering occurrence.

## Data availability statement

The original contributions presented in the study are included in the article/**Supplementary Material**. Further inquiries can be directed to the corresponding author.

## Author contributions

WL: Conceptualization, Methodology, Writing – original draft, Writing – review & editing, Formal Analysis. SQ: Data curation, Formal Analysis, Methodology, Writing – original draft. XC: Conceptualization, Funding acquisition, Supervision, Writing – review & editing, Methodology.
