# Supplementary material for: Daylength predominates the bud growth initiation of winter deciduous forest trees in the monsoon region of China
Source: Front Plant Sci. 2024 Jan 11;14:1327509. doi: 10.3389/fpls.2023.1327509 (PMC10808619; doi:10.3389/fpls.2023.1327509)
Supplement: Supplementary file 1 [file DataSheet_1.pdf]

Title: Daylength predominates the bud growth initiation of winter deciduous forest trees in the monsoon region of China

Authors: Weiguang Lang[1], Siwei Qian[1], Xiaoqiu Chen[1*],

Affiliations:
[1]College of Urban and Environmental Sciences, Laboratory for Earth Surface Processes of the Ministry of Education, Peking University, Beijing, China.
[*]Corresponding author. Email: *cxq@pku.edu.cn*

Supplementary materials:
Text S1
Table S1

Text S1. Brief introduction on three existing process-based models

In this study, we compared our revised phenological model (TPForc model) with three commonly-used one-phase models, namely, the UniForc model, the Photothermal model and the M1 model. These three models share a common framework. Specifically, the basic hypothesis is that the bud growth state (S_f) in a specific date is the accumulation of daily growth rate (R_f) from the start date (D_{start}) to the date. When the growth state (S_f) reaches the critical value (F^*) on date D_s (day of year), the spring phenology (leaf unfolding/flowering) will occur [Eq. (1)].

$$S_f = \sum_{t=D_{start}}^{D_s} R_f(T_t) = F^* \qquad (1)$$

where D_{start} was optimized as a fixed date after the 1st January or the previous winter solstice. The differences among these models are the different drivers of bud growth rate. Details are as follows.

In UniForc model, the daily growth rate (R_f) is calculated as a sigmoid function of daily mean air temperature (T_t) (Eq.2).

$$R_f(T_t) = \frac{1}{1+e^{f_a(T_t-f_b)}} \qquad (2)$$

where f_a and f_b are free parameters with $f_a < 0$ and $f_b > 0$. Therefore, the UniForc model contains four fitted parameters: D_{start}, f_a, f_b and F^*. D_{start} was optimized as a fixed date after the 1st January.

In Photothermal model, the daily growth rate is determined by the mean air temperature (T_t) and the daylength (DL) (Eq.3).

$$R_f(T_t) = \max(0, \mathrm{T}_t - T_b) \times \frac{DL}{24h} \qquad (3)$$

where T_b is the base temperature for bud growth. Therefore, the Photothermal model contains three fitted parameters: D_{start}, T_b and F^*.

In M1 model, the daily growth rate (R_f) is calculated as a sigmoid function of daily mean air temperature (T_t) and daylength (DL) (Eq.5).

$$R_f(T_t) = \max(0, \mathrm{T}_t - T_b) \times \left(\frac{DL}{10h}\right)^k \qquad (5)$$

where T_b is the base temperature for bud growth. Therefore, M1 model contains four fitted parameters: D_{start}, T_b, k and F^*.

To compare with our revised model, we optimized the D_{start} in Photothermal and M1 models as a fixed date after the previous winter solstice.

Table S1. Statistics on the optimum model types and performances for each time series. Climatic zones I, II, III, IV, V and VI denote middle temperate zone, warm temperate zone, north subtropical zone, middle subtropical zone, south subtropical zone and north tropical zone, respectively.

Phenology event	Site ID	Shortest daylength (hours)	Longest daylength (hours)	Species name	Optimum model	Climatic zone	RMSE (days)	NSE	r	VRMSE (days)
Leaf unfolding	50639	8.4	16	U. pumila	TPForcp	I	4.8	0.47	0.7	5.6
Leaf unfolding	50844	8.6	15.8	U. pumila	TPForct	I	2.6	0.64	0.81	2.8
Leaf unfolding	50936	8.7	15.7	U. pumila	TPForct	I	3	0.78	0.89	3.2
Leaf unfolding	54064	8.8	15.5	U. pumila	TPForcp	I	3	0.54	0.74	3.1
Leaf unfolding	54186	9	15.4	U. pumila	TPForct	I	3.6	0.42	0.69	5.1
Leaf unfolding	54266	9.1	15.3	U. pumila	TPForcp	I	3	0.72	0.85	4.4
Leaf unfolding	54273	9	15.4	U. pumila	TPForcp	I	4.6	0.37	0.61	5.9
Leaf unfolding	54333	9.1	15.2	U. pumila	TPForcp	I	7.4	0.22	0.48	8.7
Leaf unfolding	54353	9.1	15.2	U. pumila	TPForct	I	4.9	0.46	0.68	5.2
Leaf unfolding	52895	9.7	14.7	U. pumila	TPForct	II	3.6	0.66	0.81	5.7
Leaf unfolding	53646	9.5	14.8	U. pumila	TPForcp	II	3.6	0.23	0.46	4.5
Leaf unfolding	53754	9.6	14.8	U. pumila	TPForcp	II	4.8	0.57	0.76	6.8
Leaf unfolding	53817	9.7	14.6	U. pumila	TPForcp	II	4.5	0.62	0.79	5.8
Leaf unfolding	53845	9.7	14.7	U. pumila	TPForcp	II	6	0.53	0.73	5.5
Leaf unfolding	53986	9.8	14.5	U. pumila	TPForcp	II	4.9	0.22	0.47	5.5
Leaf unfolding	54324	9.2	15.2	U. pumila	TPForcp	II	3.2	0.67	0.82	3.6
Leaf unfolding	54326	9.2	15.2	U. pumila	TPForcp	II	7.5	0.44	0.69	7.7
Leaf unfolding	54405	9.3	15.1	U. pumila	TPForct	II	2.2	0.6	0.78	3.4
Leaf unfolding	54518	9.4	14.9	U. pumila	TPForcp	II	1.9	0.49	0.71	3.3
Leaf unfolding	54525	9.4	15	U. pumila	TPForcp	II	2.7	0.5	0.71	4.4
Leaf unfolding	56093	9.8	14.5	U. pumila	TPForcp	II	4.3	0.38	0.62	3.8
Leaf unfolding	58005	9.8	14.5	U. pumila	TPForcp	II	4.1	0.69	0.84	6.3
Leaf unfolding	57178	10	14.3	U. pumila	TPForcp	III	5.5	0.23	0.49	5.8
Leaf unfolding	57245	10	14.3	U. pumila	TPForcp	III	1.9	0.67	0.81	2.9
Leaf unfolding	57251	10	14.3	U. pumila	TPForcp	III	7.5	0.19	0.47	10.4
Leaf unfolding	57297	10	14.3	U. pumila	TPForcp	III	4.6	0.76	0.88	5.5
Leaf unfolding	57482	10.1	14.2	U. pumila	TPForcp	III	6.4	0.01	0.22	6.8
Leaf unfolding	58203	10	14.3	U. pumila	TPForcp	III	4.7	0.48	0.7	5.3
Leaf unfolding	50353	7.8	16.7	S. matsudana	TPForct	I	1.6	0.63	0.85	1.6
Leaf unfolding	50639	8.4	16	S. matsudana	TPForcp	I	3	0.58	0.74	3.7
Leaf unfolding	50873	8.5	15.9	S. matsudana	TPForct	I	6.6	0.46	0.75	7.3
Leaf unfolding	50888	8.6	15.8	S. matsudana	TPForct	I	5.6	0.47	0.69	9.6
Leaf unfolding	50936	8.7	15.7	S. matsudana	TPForct	I	5.8	0.44	0.7	10.3
Leaf unfolding	50949	8.7	15.6	S. matsudana	TPForcp	I	3.9	0.58	0.75	4.9
Leaf unfolding	50953	8.7	15.7	S. matsudana	TPForcp	I	5.8	0.11	0.34	6.6
Leaf unfolding	50983	8.7	15.7	S. matsudana	TPForcp	I	4.7	0.53	0.72	5.3
Leaf unfolding	54266	9.1	15.3	S. matsudana	TPForct	I	2.1	0.86	0.92	5.1
Leaf unfolding	54273	9	15.4	S. matsudana	TPForct	I	4.1	0.79	0.89	7.4

Leaf unfolding	54349	9.2	15.2	S. matsudana	TPForcp	I	3.4	0.59	0.77	4.6
Leaf unfolding	54353	9.1	15.2	S. matsudana	TPForct	I	5.3	0.55	0.76	7.9
Leaf unfolding	52984	9.7	14.6	S. matsudana	TPForcp	II	3.4	0.4	0.59	4.2
Leaf unfolding	53564	9.4	14.9	S. matsudana	TPForcp	II	4	0.42	0.64	4.6
Leaf unfolding	53594	9.4	15	S. matsudana	TPForcp	II	2.5	0.44	0.64	3.9
Leaf unfolding	53646	9.5	14.8	S. matsudana	TPForct	II	3.2	0.66	0.85	5
Leaf unfolding	53845	9.7	14.7	S. matsudana	TPForcp	II	6.8	0.21	0.47	8.2
Leaf unfolding	53853	9.6	14.7	S. matsudana	TPForcp	II	3.7	0.31	0.57	3.1
Leaf unfolding	53863	9.6	14.7	S. matsudana	TPForct	II	4.4	0.38	0.65	6
Leaf unfolding	53882	9.7	14.6	S. matsudana	TPForct	II	5.3	0.29	0.6	6.5
Leaf unfolding	53942	9.7	14.6	S. matsudana	TPForcp	II	8.3	0.32	0.58	9.9
Leaf unfolding	53959	9.8	14.5	S. matsudana	TPForcp	II	5	0.68	0.84	6.8
Leaf unfolding	54324	9.2	15.2	S. matsudana	TPForct	II	4.2	0.56	0.77	5.4
Leaf unfolding	54326	9.2	15.2	S. matsudana	TPForct	II	6.2	0.65	0.84	6.4
Leaf unfolding	54405	9.3	15.1	S. matsudana	TPForcp	II	1.8	0.84	0.9	2.7
Leaf unfolding	54525	9.4	15	S. matsudana	TPForcp	II	3.8	0.58	0.76	5.4
Leaf unfolding	54534	9.4	15	S. matsudana	TPForcp	II	2	0.76	0.86	2.1
Leaf unfolding	54725	9.6	14.8	S. matsudana	TPForcp	II	4	0.76	0.86	4.6
Leaf unfolding	54843	9.6	14.7	S. matsudana	TPForcp	II	4.8	0.17	0.43	5.8
Leaf unfolding	56093	9.8	14.5	S. matsudana	TPForct	II	4.8	0.57	0.76	5.6
Leaf unfolding	57014	9.8	14.5	S. matsudana	TPForcp	II	2.4	0.85	0.93	3.3
Leaf unfolding	57025	9.8	14.5	S. matsudana	TPForcp	II	2.1	0.93	0.97	3
Leaf unfolding	57030	9.8	14.5	S. matsudana	TPForcp	II	5.8	0.59	0.78	7.7
Leaf unfolding	57251	10	14.3	S. matsudana	TPForcp	III	5.6	0.54	0.77	8.4
Leaf unfolding	57476	10.2	14.1	S. matsudana	TPForcp	III	5.9	0.67	0.82	7.7
Leaf unfolding	57483	10.2	14.1	S. matsudana	TPForcp	III	6.9	0.5	0.7	8.1
Leaf unfolding	57493	10.2	14.1	S. matsudana	TPForcp	III	4.9	0.69	0.83	6.3
Leaf unfolding	57494	10.2	14.1	S. matsudana	TPForcp	III	8.1	0.44	0.69	10.8
Leaf unfolding	57581	10.2	14.1	S. matsudana	TPForcp	III	3.6	0.68	0.82	4
Leaf unfolding	57662	10.3	14	S. matsudana	TPForcp	III	5.4	0.51	0.72	6.3
Leaf unfolding	58208	10	14.3	S. matsudana	TPForcp	III	4	0.85	0.93	6.2
Leaf unfolding	56751	10.5	13.7	S. matsudana	TPForcp	IV	4.3	0.53	0.71	5.6
Leaf unfolding	57083	9.8	14.5	M. azedarach	TPForcp	II	3.1	0.57	0.75	3.6
Leaf unfolding	58038	9.9	14.4	M. azedarach	TPForcp	II	3.7	0.57	0.76	5.9
Leaf unfolding	58102	9.9	14.4	M. azedarach	TPForct	II	4.3	0.6	0.78	5.3
Leaf unfolding	58122	9.9	14.4	M. azedarach	TPForcp	II	3.4	0.54	0.74	3.6
Leaf unfolding	57290	10	14.3	M. azedarach	TPForcp	III	4.6	0.58	0.77	4.9
Leaf unfolding	57297	10	14.3	M. azedarach	TPForcp	III	4.5	0.69	0.83	5.1
Leaf unfolding	57378	10.1	14.2	M. azedarach	TPForcp	III	1.6	0.83	0.92	4.2
Leaf unfolding	57493	10.2	14.1	M. azedarach	TPForcp	III	3.3	0.58	0.79	5.8
Leaf unfolding	57662	10.3	14	M. azedarach	TPForcp	III	6.6	0.59	0.76	8
Leaf unfolding	58158	10	14.4	M. azedarach	TPForcp	III	1.7	0.75	0.85	2.2
Leaf unfolding	58203	10	14.3	M. azedarach	TPForct	III	4.9	0.31	0.54	5.9
Leaf unfolding	58236	10	14.3	M. azedarach	TPForct	III	3.5	0.64	0.8	3.7

Leaf unfolding	58252	10	14.3	M. azedarach	TPForcp	III	3	0.79	0.87	3.6
Leaf unfolding	58255	10	14.3	M. azedarach	TPForct	III	3.4	0.61	0.81	5.4
Leaf unfolding	57523	10.2	14.1	M. azedarach	TPForcp	IV	5	0.52	0.73	6.3
Leaf unfolding	57679	10.3	13.9	M. azedarach	TPForcp	IV	6.1	0.39	0.6	8.9
Leaf unfolding	57696	10.3	14	M. azedarach	TPForct	IV	3.7	0.84	0.92	6.5
Leaf unfolding	57789	10.4	13.9	M. azedarach	TPForcp	IV	4.6	0.82	0.91	5.7
Leaf unfolding	57889	10.5	13.8	M. azedarach	TPForcp	IV	3.7	0.76	0.9	9.7
Leaf unfolding	57947	10.6	13.7	M. azedarach	TPForcp	IV	6.9	0.75	0.87	9.1
Leaf unfolding	57958	10.6	13.7	M. azedarach	TPForcp	IV	4.3	0.87	0.94	5.7
Leaf unfolding	58608	10.4	13.9	M. azedarach	TPForcp	IV	4.5	0.44	0.72	8.4
Leaf unfolding	58718	10.4	13.9	M. azedarach	TPForcp	IV	4.2	0.79	0.9	6.1
Leaf unfolding	58731	10.4	13.9	M. azedarach	TPForcp	IV	3	0.81	0.92	4.8
Leaf unfolding	58806	10.5	13.8	M. azedarach	TPForcp	IV	4.2	0.84	0.91	5.8
Leaf unfolding	59058	10.6	13.6	M. azedarach	TPForcp	IV	6.1	0.73	0.86	8.9
Leaf unfolding	59072	10.6	13.7	M. azedarach	TPForcp	IV	5.5	0.77	0.88	6.2
Leaf unfolding	59082	10.6	13.7	M. azedarach	TPForcp	IV	8.4	0.42	0.66	8.3
Leaf unfolding	59092	10.6	13.7	M. azedarach	TPForcp	IV	6.1	0.69	0.84	8.3
Leaf unfolding	59023	10.6	13.7	M. azedarach	TPForcp	V	5.5	0.66	0.83	7.3
Leaf unfolding	59037	10.7	13.6	M. azedarach	TPForcp	V	6.4	0.63	0.8	7.4
Leaf unfolding	59117	10.6	13.6	M. azedarach	TPForcp	V	5.2	0.56	0.77	5.8
Leaf unfolding	59211	10.7	13.6	M. azedarach	TPForcp	V	6	0.67	0.83	6.7
Leaf unfolding	59218	10.7	13.6	M. azedarach	TPForcp	V	3.8	0.85	0.92	5.6
Leaf unfolding	59254	10.7	13.6	M. azedarach	TPForcp	V	7.4	0.52	0.72	9.6
Leaf unfolding	59278	10.7	13.6	M. azedarach	TPForcp	V	7.2	0.59	0.77	7.1
Leaf unfolding	59293	10.7	13.6	M. azedarach	TPForcp	V	9.1	0.3	0.56	11.1
Leaf unfolding	59431	10.7	13.5	M. azedarach	TPForcp	V	6.6	0.7	0.84	7.3
Leaf unfolding	59446	10.8	13.5	M. azedarach	TPForcp	V	4.2	0.81	0.9	5.4
Leaf unfolding	59453	10.7	13.5	M. azedarach	TPForcp	V	6	0.63	0.81	8.4
Leaf unfolding	59485	10.8	13.5	M. azedarach	TPForcp	V	8.5	0.36	0.6	10.9
Leaf unfolding	59632	10.8	13.5	M. azedarach	TPForcp	V	7.1	0.51	0.72	10.6
Leaf unfolding	59663	10.8	13.5	M. azedarach	TPForcp	V	10.3	0.26	0.54	13.4
Leaf unfolding	59754	10.9	13.4	M. azedarach	TPForcp	VI	5.7	0.33	0.61	6.6
Leaf unfolding	59845	11	13.3	M. azedarach	TPForcp	VI	9.3	0.49	0.71	14.1
Leaf unfolding	59849	11	13.3	M. azedarach	TPForcp	VI	9	0.35	0.63	10.7
Leaf unfolding	59954	11	13.2	M. azedarach	TPForcp	VI	4.2	0.17	0.46	3.9
Leaf unfolding	56763	10.5	13.7	B. ceiba	TPForcp	IV	7.8	0.52	0.72	8.6
Leaf unfolding	56966	10.7	13.6	B. ceiba	TPForcp	V	4	0.5	0.71	4.6
Leaf unfolding	59211	10.7	13.6	B. ceiba	TPForcp	V	5.3	0.78	0.89	6.9
Leaf unfolding	59218	10.7	13.6	B. ceiba	TPForcp	V	8.3	0.54	0.76	10.3
Leaf unfolding	59254	10.7	13.6	B. ceiba	TPForcp	V	7.3	0.57	0.77	8.4
Leaf unfolding	59278	10.7	13.6	B. ceiba	TPForcp	V	8.9	0.68	0.83	10.3
Leaf unfolding	59431	10.7	13.5	B. ceiba	TPForcp	V	4.9	0.76	0.88	5.6
Leaf unfolding	59446	10.8	13.5	B. ceiba	TPForcp	V	5	0.81	0.91	5.9
Leaf unfolding	59453	10.7	13.5	B. ceiba	TPForcp	V	7.9	0.73	0.86	9.5

Leaf unfolding	59485	10.8	13.5	B. ceiba	TPForct	V	9	0.47	0.7	10.9
Leaf unfolding	59632	10.8	13.5	B. ceiba	TPForcp	V	3.2	0.96	0.98	5.9
Leaf unfolding	59663	10.8	13.5	B. ceiba	TPForcp	V	6.9	0.29	0.59	7.5
Leaf unfolding	59849	11	13.3	B. ceiba	TPForcp	VI	8.6	0.3	0.55	9.8
Flowering	50639	8.4	16	U. pumila	TPForcp	I	11.9	0.19	0.43	13.5
Flowering	50844	8.6	15.8	U. pumila	TPForcp	I	2.9	0.61	0.77	3.4
Flowering	50936	8.7	15.7	U. pumila	TPForct	I	3.3	0.74	0.86	4.9
Flowering	54064	8.8	15.5	U. pumila	TPForcp	I	5	0.36	0.62	6.8
Flowering	54186	9	15.4	U. pumila	TPForct	I	7.6	0.42	0.65	8.6
Flowering	54266	9.1	15.3	U. pumila	TPForct	I	3.6	0.63	0.81	5.3
Flowering	54273	9	15.4	U. pumila	TPForcp	I	5.1	0.11	0.4	6.7
Flowering	54333	9.1	15.2	U. pumila	TPForct	I	7.7	0.55	0.75	11.1
Flowering	54353	9.1	15.2	U. pumila	TPForcp	I	7.3	0.35	0.61	8.9
Flowering	52895	9.7	14.7	U. pumila	TPForcp	II	6.8	0.23	0.51	8.2
Flowering	53646	9.5	14.8	U. pumila	TPForcp	II	3.7	0.35	0.64	8.7
Flowering	53754	9.6	14.8	U. pumila	TPForct	II	4.4	0.76	0.89	5.9
Flowering	53817	9.7	14.6	U. pumila	TPForcp	II	6.3	0.39	0.63	6.4
Flowering	53845	9.7	14.7	U. pumila	TPForcp	II	9.6	0.53	0.78	10.6
Flowering	53986	9.8	14.5	U. pumila	TPForct	II	8.5	0.51	0.72	10.6
Flowering	54324	9.2	15.2	U. pumila	TPForct	II	6.5	0.37	0.62	8.6
Flowering	54326	9.2	15.2	U. pumila	TPForcp	II	7.6	0.32	0.58	10.1
Flowering	54405	9.3	15.1	U. pumila	TPForcp	II	1.4	0.85	0.91	1.4
Flowering	54518	9.4	14.9	U. pumila	TPForcp	II	4.3	0.51	0.72	6.2
Flowering	54525	9.4	15	U. pumila	TPForcp	II	5.2	0.13	0.47	6
Flowering	56093	9.8	14.5	U. pumila	TPForct	II	4.9	0.37	0.61	7.6
Flowering	58005	9.8	14.5	U. pumila	TPForcp	II	10	0.46	0.75	9.4
Flowering	57178	10	14.3	U. pumila	TPForcp	III	12.9	0.32	0.57	18.1
Flowering	57245	10	14.3	U. pumila	TPForcp	III	2.5	0.6	0.77	4.2
Flowering	57251	10	14.3	U. pumila	TPForcp	III	14.1	0.33	0.62	17.8
Flowering	57297	10	14.3	U. pumila	TPForcp	III	6.5	0.46	0.73	9.2
Flowering	57482	10.1	14.2	U. pumila	TPForcp	III	7.5	0.08	0.44	7.7
Flowering	58203	10	14.3	U. pumila	TPForcp	III	6.6	0.34	0.55	6.6
Flowering	50353	7.8	16.7	S. matsudana	TPForcp	I	2.3	0.62	0.74	3.9
Flowering	50639	8.4	16	S. matsudana	TPForct	I	12.9	0.24	0.5	15.5
Flowering	50873	8.5	15.9	S. matsudana	TPForct	I	6.1	0.45	0.67	8.6
Flowering	50888	8.6	15.8	S. matsudana	TPForct	I	4.9	0.57	0.8	7.4
Flowering	50936	8.7	15.7	S. matsudana	TPForct	I	2.2	0.88	0.94	3.5
Flowering	50949	8.7	15.6	S. matsudana	TPForct	I	7.2	0.51	0.72	10.5
Flowering	50953	8.7	15.7	S. matsudana	TPForcp	I	2.9	0.21	0.5	3.4
Flowering	50983	8.7	15.7	S. matsudana	TPForcp	I	4.4	0.22	0.46	6.3
Flowering	54266	9.1	15.3	S. matsudana	TPForcp	I	4.5	0.68	0.82	8.1
Flowering	54273	9	15.4	S. matsudana	TPForct	I	5.5	0.55	0.77	9.3
Flowering	54349	9.2	15.2	S. matsudana	TPForcp	I	3.9	0.6	0.77	5.5
Flowering	54353	9.1	15.2	S. matsudana	TPForct	I	3.4	0.72	0.86	4.8

Flowering	52984	9.7	14.6	S. matsudana	TPForcp	II	12.3	0.06	0.26	12.1
Flowering	53564	9.4	14.9	S. matsudana	TPForct	II	6.9	0.49	0.73	7.6
Flowering	53594	9.4	15	S. matsudana	TPForcp	II	2.1	0.49	0.72	3.1
Flowering	53646	9.5	14.8	S. matsudana	TPForcp	II	3.8	0.26	0.46	7.1
Flowering	53845	9.7	14.7	S. matsudana	TPForcp	II	8.2	0.44	0.72	8.3
Flowering	53853	9.6	14.7	S. matsudana	TPForcp	II	4.9	0.28	0.52	5.7
Flowering	53863	9.6	14.7	S. matsudana	TPForct	II	7	0.4	0.64	12
Flowering	53882	9.7	14.6	S. matsudana	TPForcp	II	2.5	0.47	0.73	4.9
Flowering	53942	9.7	14.6	S. matsudana	TPForcp	II	4.9	0.22	0.53	6.7
Flowering	53959	9.8	14.5	S. matsudana	TPForct	II	5.8	0.68	0.83	6
Flowering	54324	9.2	15.2	S. matsudana	TPForct	II	5.9	0.48	0.7	7.9
Flowering	54326	9.2	15.2	S. matsudana	TPForct	II	7.8	0.39	0.64	10.6
Flowering	54405	9.3	15.1	S. matsudana	TPForct	II	1.4	0.9	0.96	3.4
Flowering	54525	9.4	15	S. matsudana	TPForct	II	4.7	0.68	0.83	7.3
Flowering	54534	9.4	15	S. matsudana	TPForcp	II	3.3	0.63	0.81	5.1
Flowering	54725	9.6	14.8	S. matsudana	TPForct	II	4.2	0.67	0.82	4.9
Flowering	54843	9.6	14.7	S. matsudana	TPForcp	II	5.7	0.17	0.38	5.3
Flowering	56093	9.8	14.5	S. matsudana	TPForct	II	3.5	0.68	0.83	4.5
Flowering	57014	9.8	14.5	S. matsudana	TPForcp	II	3.2	0.83	0.92	5.7
Flowering	57025	9.8	14.5	S. matsudana	TPForcp	II	4.7	0.66	0.84	6.3
Flowering	57030	9.8	14.5	S. matsudana	TPForcp	II	6.5	0.44	0.67	5.2
Flowering	57251	10	14.3	S. matsudana	TPForcp	III	5.7	0.45	0.67	6.5
Flowering	57476	10.2	14.1	S. matsudana	TPForcp	III	7.2	0.26	0.48	8.7
Flowering	57483	10.2	14.1	S. matsudana	TPForct	III	10.1	0.43	0.67	12.7
Flowering	57493	10.2	14.1	S. matsudana	TPForcp	III	4	0.76	0.87	5.3
Flowering	57494	10.2	14.1	S. matsudana	TPForcp	III	10.5	0.46	0.68	15.3
Flowering	57581	10.2	14.1	S. matsudana	TPForcp	III	4.3	0.51	0.73	6.6
Flowering	57662	10.3	14	S. matsudana	TPForct	III	6.9	0.53	0.74	10.4
Flowering	58208	10	14.3	S. matsudana	TPForcp	III	5.1	0.54	0.73	7.2
Flowering	56751	10.5	13.7	S. matsudana	TPForcp	IV	4.6	0.58	0.76	6.4
Flowering	57083	9.8	14.5	M. azedarach	TPForcp	II	2.8	0.64	0.8	3.4
Flowering	58038	9.9	14.4	M. azedarach	TPForcp	II	4.1	0.34	0.61	5.5
Flowering	58102	9.9	14.4	M. azedarach	TPForcp	II	6.7	0.09	0.33	8.1
Flowering	58122	9.9	14.4	M. azedarach	TPForcp	II	2.8	0.72	0.86	3.2
Flowering	57290	10	14.3	M. azedarach	TPForcp	III	4.8	0.35	0.58	6.3
Flowering	57297	10	14.3	M. azedarach	TPForcp	III	3	0.79	0.89	3.7
Flowering	57378	10.1	14.2	M. azedarach	TPForcp	III	7.6	0.52	0.74	9.6
Flowering	57493	10.2	14.1	M. azedarach	TPForcp	III	5.9	0.46	0.7	5.8
Flowering	57662	10.3	14	M. azedarach	TPForcp	III	6.9	0.2	0.49	8.3
Flowering	58158	10	14.4	M. azedarach	TPForcp	III	2.4	0.49	0.7	3
Flowering	58203	10	14.3	M. azedarach	TPForcp	III	5.1	0.48	0.7	4.5
Flowering	58236	10	14.3	M. azedarach	TPForcp	III	2.7	0.7	0.83	3.5
Flowering	58252	10	14.3	M. azedarach	TPForct	III	2.6	0.84	0.92	3.4
Flowering	58255	10	14.3	M. azedarach	TPForct	III	4.1	0.72	0.86	5.4

Flowering	57523	10.2	14.1	M. azedarach	TPForcp	IV	5.7	0.35	0.61	7.9
Flowering	57679	10.3	13.9	M. azedarach	TPForcp	IV	4.1	0.68	0.83	6.5
Flowering	57696	10.3	14	M. azedarach	TPForcp	IV	1.8	0.85	0.92	2.5
Flowering	57789	10.4	13.9	M. azedarach	TPForcp	IV	2.7	0.85	0.92	4.1
Flowering	57889	10.5	13.8	M. azedarach	TPForcp	IV	6.4	0.22	0.57	9.1
Flowering	57947	10.6	13.7	M. azedarach	TPForcp	IV	4.5	0.57	0.74	6.5
Flowering	57958	10.6	13.7	M. azedarach	TPForct	IV	3.3	0.87	0.93	3.9
Flowering	58608	10.4	13.9	M. azedarach	TPForcp	IV	5.2	0.27	0.5	8.8
Flowering	58718	10.4	13.9	M. azedarach	TPForcp	IV	2.3	0.85	0.92	2.2
Flowering	58731	10.4	13.9	M. azedarach	TPForcp	IV	3.3	0.59	0.78	5.4
Flowering	58806	10.5	13.8	M. azedarach	TPForcp	IV	2.9	0.82	0.92	3.5
Flowering	59058	10.6	13.6	M. azedarach	TPForcp	IV	4.1	0.77	0.88	5.1
Flowering	59072	10.6	13.7	M. azedarach	TPForcp	IV	2.4	0.95	0.98	4.4
Flowering	59082	10.6	13.7	M. azedarach	TPForcp	IV	3.1	0.88	0.93	4.9
Flowering	59092	10.6	13.7	M. azedarach	TPForcp	IV	3.1	0.92	0.96	4.7
Flowering	59023	10.6	13.7	M. azedarach	TPForcp	V	5.1	0.65	0.81	8.8
Flowering	59037	10.7	13.6	M. azedarach	TPForcp	V	3.8	0.87	0.93	5.5
Flowering	59117	10.6	13.6	M. azedarach	TPForcp	V	6.9	0.4	0.64	9.8
Flowering	59211	10.7	13.6	M. azedarach	TPForcp	V	4.5	0.75	0.87	6.1
Flowering	59218	10.7	13.6	M. azedarach	TPForcp	V	9.2	0.55	0.75	11.9
Flowering	59254	10.7	13.6	M. azedarach	TPForcp	V	4.9	0.69	0.83	6
Flowering	59278	10.7	13.6	M. azedarach	TPForcp	V	6.8	0.71	0.84	7.9
Flowering	59293	10.7	13.6	M. azedarach	TPForcp	V	8	0.59	0.76	9.5
Flowering	59431	10.7	13.5	M. azedarach	TPForcp	V	4.8	0.82	0.9	5.3
Flowering	59446	10.8	13.5	M. azedarach	TPForcp	V	5	0.8	0.9	6.8
Flowering	59453	10.7	13.5	M. azedarach	TPForcp	V	4.1	0.87	0.93	4.4
Flowering	59485	10.8	13.5	M. azedarach	TPForcp	V	7.6	0.39	0.63	12.4
Flowering	59632	10.8	13.5	M. azedarach	TPForcp	V	4	0.88	0.94	3.5
Flowering	59663	10.8	13.5	M. azedarach	TPForcp	V	6.5	0.5	0.72	7.8
Flowering	59754	10.9	13.4	M. azedarach	TPForcp	VI	3.8	0.56	0.77	6.5
Flowering	59845	11	13.3	M. azedarach	TPForcp	VI	4	0.45	0.65	4.2
Flowering	59849	11	13.3	M. azedarach	TPForcp	VI	6.9	0.15	0.41	7.9
Flowering	59954	11	13.2	M. azedarach	TPForcp	VI	4.1	0.27	0.51	6.1
Flowering	56763	10.5	13.7	B. ceiba	TPForcp	IV	6.3	0.6	0.82	12.2
Flowering	56966	10.7	13.6	B. ceiba	TPForcp	V	9.1	0.14	0.4	13.5
Flowering	59211	10.7	13.6	B. ceiba	TPForcp	V	11.7	0.32	0.64	17.2
Flowering	59218	10.7	13.6	B. ceiba	TPForcp	V	10.3	0.67	0.85	15.4
Flowering	59254	10.7	13.6	B. ceiba	TPForcp	V	11.1	0.51	0.74	15.3
Flowering	59278	10.7	13.6	B. ceiba	TPForcp	V	6.5	0.45	0.69	8.6
Flowering	59431	10.7	13.5	B. ceiba	TPForcp	V	8	0.54	0.75	12.8
Flowering	59446	10.8	13.5	B. ceiba	TPForcp	V	8.3	0.6	0.77	13.4
Flowering	59453	10.7	13.5	B. ceiba	TPForcp	V	15.7	0.37	0.61	19
Flowering	59485	10.8	13.5	B. ceiba	TPForcp	V	8.4	0.3	0.54	10.3
Flowering	59632	10.8	13.5	B. ceiba	TPForcp	V	8.7	0.72	0.88	13.6

Flowering	59663	10.8	13.5	B. ceiba	TPForcp	V	6.3	0.28	0.53	5.2
Flowering	59849	11	13.3	B. ceiba	TPForcp	VI	7.8	0.12	0.36	7.6
